# Enhancement of laryngeal contrasts in non-native English clear speech: a comparison between L2-immersed sequential bilinguals and L1-immersed speakers

**DOI:** 10.3389/fnhum.2024.1413886

**Published:** 2024-09-13

**Authors:** Ye Jee Jung, Olga Dmitrieva

**Affiliations:** ^1^Department of Speech-Language Pathology and Audiology, Hunter College, City University of New York, New York, NY, United States; ^2^Department of Linguistics, Purdue University, West Lafayette, IN, United States

**Keywords:** English, Korean, hyperarticulation, clear speech, laryngeal contrasts

## Abstract

Clear speech, a speaking style used to mitigate communicative circumstances affecting the transmission or decoding of speech signal, often involves the enhancement of language-specific phonological contrasts, including laryngeal contrasts. This study investigates the role of language dominance in the implementation of language-specific laryngeal contrasts in L2 clear speech. Two groups of Korean-English speakers (L1 Korean) were tested: a relatively less Korean-dominant L2-immersed group of sequential bilinguals (*N* = 30) and a strongly Korean-dominant L1-immersed group (*N* = 30), with dominance assessed based on the results of the Bilingual Language Profile. Participants read a set of English minimal pairs differing in the voicing of word-initial stops (e.g., *tab* vs. *dab*), and their acoustic enhancement strategies were compared with those of native English speakers (*N* = 20). As correlates of the English laryngeal contrast, voice onset time (VOT) and onset f0 were measured. Results showed that both bilingual groups enhanced English laryngeal contrast in clear speech via voiceless VOT lengthening, similarly to native English speakers, but to a smaller extent than native speakers. Both bilingual groups also implemented a greater degree of onset f0 difference between voiced and voiced English stops than native English speakers did, although no enhancement of this parameter was observed in their clear speech. Surprisingly, no significant differences were found between L2- and L1-immersed speakers, suggesting a lack of language immersion effect on the acoustic enhancement strategies in L2 clear speech. We discuss possible explanations for this finding and propose directions for future research.

## 1 Introduction

The ability to adopt a situation-appropriate speaking style, including implementing the necessary acoustic modifications in speech, forms part of one’s linguistic abilities (Lindblom, 1990). Clear speech is an example of a listener-accommodating speaking style aiming at increasing the intelligibility of speech in unfavorable circumstances, such as in the presence of background noise or when addressing hearing-impaired interlocutors. It is well established that clear speech produced by native-speaking populations is acoustically different from casual speech in both suprasegmental and segmental domains. Suprasegmental characteristics of clear speech typically include slower speaking rate, increased mean f0 and f0 range, and increased intensity (e.g., Picheny et al., 1986; Krause and Braida, 2004). Segmental features of clear speech related to modifications of acoustic properties of vowels and consonants include, for example, longer vowel duration, emphasized vowel formants leading to the expansion of the vowel space area, and longer voice onset time (VOT) of voiceless stops (e.g., Ferguson and Kewley-Port, 2007; Smiljanic and Bradlow, 2011). Moreover, as a result of these modifications, certain phonological contrasts, such as the contrast between English voiceless and voiced stops, are phonetically enhanced in clear speech (Picheny et al., 1986; Jung and Dmitrieva, 2023).

The bulk of previous literature has investigated clear speech produced by native, monolingual speakers of English (e.g., Picheny et al., 1986; Krause and Braida, 2004; Smiljanic and Bradlow, 2005). More recently, a small but growing body of research began to examine English clear speech produced by non-native populations, such as native Finnish (Granlund et al., 2012), Cantonese (Li and So, 2006), Mandarin (Kato and Baese-Berk, 2022), and Korean (Jung and Dmitrieva, 2023) speakers.

The need to investigate non-native clear speech is tied to a simple but important fact that spoken interactions between native and non-native speakers are becoming more common than ever, including in high-stakes settings where clarity of communication is critical, such as aviation, science and technology, national security, and healthcare. It has been documented that non-native speech is often less intelligible than native speech (e.g., Van Wijngaarden, 2001; McLaughlin et al., 2018). Moreover, the intelligibility disadvantage of non-native speech is compounded by the presence of extra-linguistic barriers such as ambient noise (e.g., Scharenborg and van Os, 2019). Thus, it is important to understand how non-native speakers cope with communicative disadvantages in order to increase intelligibility of their speech.

Overall, findings from the existing studies demonstrate that the nature of acoustic modifications in non-native clear speech can be comparable to that of native clear speech. In other words, non-native clear speech tends to have the same acoustic adjustments as native clear speech. For instance, in Granlund et al. (2012), late Finnish-English bilinguals showed native-like modifications of fundamental frequency (f0) median, f0 range, mean energy 1–3 kHz, and mean word duration in English clear speech. In Jung and Dmitrieva (2023), late Korean-English bilinguals made vowel duration and vowel space modifications in English clear speech that were similar to those produced by native English speakers. However, the degree of the bilingual speakers’ modification was smaller compared with native English speakers. The same pattern (i.e., a native-like adjustment of a smaller magnitude) was also observed for vowel space area that was clearly produced by Cantonese speakers of English (Li and So, 2006).

It should be noted that in those studies, non-native speakers were often proficient second language (L2) learners and were residing in an L2-immersed environment, i.e., in an English-speaking country. The most underexplored part of research on non-native clear speech production is a comparison between non-native speaker groups with different levels of L2 proficiency and exposure to L2. To date, this line of inquiry has been directly examined in only two studies (Kato and Baese-Berk, 2022, 2024).^1^ In both studies, the authors presented acoustic analyses of English clear speech produced by the three speaker groups: native English speakers, native Mandarin speakers with higher English proficiency (Mandarin-High), and native Mandarin speakers with lower English proficiency (Mandarin-Low). To make a clear distinction between the two native Mandarin speaker groups in terms of English proficiency, the authors collected information about their L2 experiences, including age of onset for English speaking, years of formal English training, length of US residence in months, and TOEFL score.

In these studies, acoustic analyses included both suprasegmental (i.e., speaking rate, mean f0, and f0 range) and segmental (i.e., vowel space and vowel duration) parameters. The between-group analyses revealed that, in general, native Mandarin-Low speakers were distinguishable from both native English speakers and native Mandarin-High speakers in that they produced a smaller degree of acoustic modifications in clear speech. For instance, native Mandarin-High speakers slowed down their speech to speak clearly to an extent similar to native English speakers. However, native Mandarin-Low speakers did not decrease their speaking rate to a comparable degree. A similar pattern was observed for other acoustic parameters, pointing to the fact that non-native speakers’ L2 proficiency had a pronounced impact on the modification strategies adopted in L2 clear speech: lower L2 proficiency resulted in clear speech enhancements that were not comparable in magnitude to those produced by native speakers and more proficient L2 speakers.

Although Kato and Baese-Berk made a distinction between the two native Mandarin speaker groups in their 2022 and 2024 studies based on a variety of factors, both groups were L2-immersed, as all native Mandarin speakers in their studies were residing in the United States at the time of participation. Due to this fact, the two speaker groups could have had a largely comparable amount of exposure to L2, at least for the duration of L2 immersion. L2 immersion provides benefits to L2 learning that are not easily obtained in an L1-immersed environment, especially with respect to spoken language (e.g., Segalowitz et al., 2004; Lord, 2010). Therefore, it could be hypothesized that were the two groups further contrasted on the basis of L2-immersion, a more profound difference in their L2 clear speech implementation could emerge. This is the hypothesis tested in the present study.

The purpose of the current study is to conduct an acoustic analysis of Korean-accented English clear speech produced by the two different Korean speaker groups: L1- and L2-immersed speakers. In addition, analysis of native English clear speech will be included. Of particular interest is how the laryngeal contrast (i.e., voiceless vs. voiced) is enhanced in English clear speech via VOT of word-initial stops and f0 at the onset of the vowel that follows the stops (onset f0, henceforth). We focus on these parameters because previous studies on native English (e.g., Picheny et al., 1986) and native Korean (Kang and Guion, 2008) clear speech demonstrated that these two acoustic parameters were modified in a different fashion in each of the two languages, which is considered a reflection of the language-specific production strategies that will be described in detail below.

In English, VOT – the temporal interval between stop release and onset of voicing (glottal pulsing) – serves as a primary acoustic parameter that makes a distinction between word-initial voiced and voiceless stops (e.g., Abramson and Lisker, 1985; Whalen et al., 1993). On the other hand, onset f0 – fundamental frequency at the onset of vowel following the stop - plays a secondary role in cuing of the English voicing contrast (e.g., Whalen et al., 1990). Nevertheless, the subtle but consistent onset f0 difference between the two stop types exists, as higher onset f0 is a correlate of voiceless stops, while voiced stops are produced with lower onset f0 (Kong and Weismer, 2010; Dmitrieva et al., 2015). As opposed to English, it has been argued that both VOT and onset f0 are crucial in marking a three-way distinction between aspirated, lenis, and fortis stops in Korean (e.g., Silva, 2006). Table 1 illustrates that each category of a Korean stop has its own unique signature in terms of the values of VOT and onset f0 (also see Kang and Guion, 2006 for additional acoustic characteristics of Korean stops). Furthermore, since aspirated and lenis stops have been merging with respect to VOT in contemporary Seoul Korean, onset f0 took on the function of a primary acoustic correlate distinguishing these categories from one another. In summary, although both VOT and onset f0 are used in the laryngeal phonology of both languages, they are used differently in demarcating laryngeal contrasts in each of the languages.

**Table 1:** VOT and Onset f0 values for each Korean stop type (based on Kang and Guion, 2006).

Stop type	Acoustic parameters	
	VOT	onset f0
Aspirated	Longest	Highest
Lenis	Longer	Lowest
Fortis	Shortest	Higher

Consequently, it is reasonable to expect that these acoustic parameters, VOT and onset f0, are modified differently in Korean and English clear speech, in accordance with their functional load in cuing the laryngeal categories in each language. This prediction is upheld in existing literature. In Korean, both onset f0 differences and VOT differences between contrasting laryngeal categories are emphasized in clear speech, with greater weight given to one or another parameter depending on the specific contrast. For example, onset f0 is enhanced for the clearly produced lenis-aspirated contrast (Kang and Guion, 2008). In contrast, English clearly produced voicing contrast emphasizes VOT differences only, but not the onset f0 differences (Jung and Dmitrieva, 2023).

Given these differences in language-specific realization and enhancement of laryngeal contrast, it is possible that L2 speakers with less L2 experience and exposure, especially those without immersive L2 experience, may rely more strongly on L1-based clear speech strategies in producing L2 clear speech, than speakers with more extensive, immersive L2 experience. The general pattern of L1 influence in L2 speech production is compatible with predictions of all prominent theories of acquisition of L2 speech, such as the revised Speech Learning Model (SLM-r; Flege and Bohn, 2021). It is based on the idea that L2 speakers establish links between perceptually similar L1 and L2 categories, and such links lead to mutual influence in speech production, with the strength of L1 influence declining with greater L2 proficiency and exposure.

We expand on this general prediction by hypothesizing that L1 influence on L2 speech production extends into the domain of accommodation in speech. Moreover, we predict that L1 influence can manifest itself in terms of preferential selection of one acoustic parameter over another for enhancement in clear speech. Specifically, we expect that L1-immersed speakers will enhance the onset f0 differences between English voicing categories to a greater extent than L2-immersed speakers, in accordance with an L1-based strategy. In addition, based on Kato and Baese-Berk (2022, 2024) we expect that clear-speech related enhancement of VOT in English voicing contrast will be smaller in magnitude in the speech of L1-immersed participants than in the speech of L2-immersed participants.

To conclude, the research question addressed in the current study is: How do L2- and L1-immersed native Korean speakers enhance the laryngeal contrast of English in clear speech in comparison with native English speakers? The hypothesis tested is: L1-immersed speakers will utilize more Korean-like strategies in enhancing English laryngeal contrast than L2-immersed speakers. Specifically, we expect that L1-immersed speakers will enhance the onset f0 differences between the voicing categories to a greater extent than L2-immersed speakers.

## 2 Methods

### 2.1 Participants

Three groups of participants were recruited for the study: monolingual English speakers (NE; *n* = 20, mean age = 24.95 years, 4 male),^2^ late sequential Korean-English bilingual immersed in the L2-environment (KE; *n* = 30, mean age = 29.73 years, 19 male), and L1-immersed Korean speakers (NK; *n* = 30, mean age = 27.67 years, 11 male). NE speakers were native speakers of Midwestern American English. All the KE speakers were residents of a midwestern region of the United States at the time of recording (mean length of residence in an English-speaking country = 3.43 years, mean age of onset of learning English = 9.07 years) The NK speakers were residing in Seoul, South Korea at the time of participation (mean length of residence in an English-speaking country = 0.00 years, mean age of onset of learning English = 8.53 years), and they spoke contemporary Seoul Korean as their native language.

**Figure 1:**
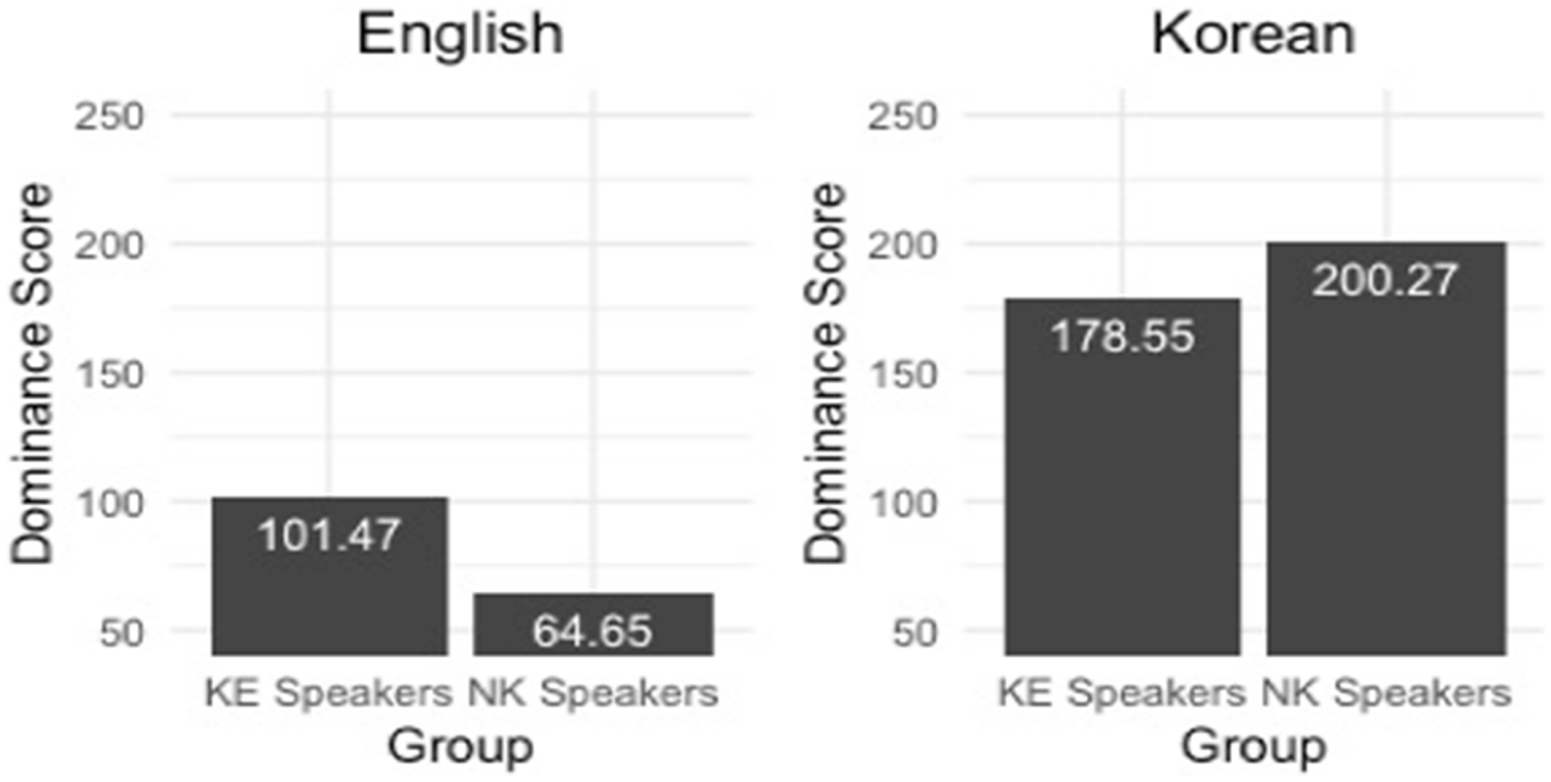
A global language score of each of the speaker groups for English (left panel) and Korean (right panel).

The analysis of the results of the Bilingual Language Profile (BLP) questionnaire (Birdsong et al., 2012) completed by the KE and the NK speakers demonstrate that the two speaker groups were different from one another with respect to a global language score^3^ calculated for each of the two languages (see Figure 1). A possible score for this domain ranges from 0 to 218, with 0 indicating a complete absence of knowledge and experience for a given language (see also Olson, 2023). The independent (i.e., two-sample) t-test was implemented in R (R Core Team, 2019) using the *t.test()* function to confirm whether the between-group difference was statistically significant for each of the two languages. The t-test results confirmed that the KE speakers had a greater global English score than the NK speakers [*t* (56) = 7.20, *p* < 0.001]. At the same time, they had a smaller global Korean score in comparison with the NK speakers [*t* (57) = −6.02, *p* < 0.001]. In other words, the KE speakers overall had a smaller Korean-English global language score difference, although both speaker groups can be categorized as Korean-dominant bilinguals, as the global language score was always greater in Korean than in English regardless of the group. The two Korean speaker groups were also significantly different from each other with respect to the sum score of both English-proficiency [*t* (54) = 3.00, *p* < 0.01] and English-attitudes modules [*t* (55) = 2.82, *p* < 0.001]. That is, the KE speakers had a higher self-reported English proficiency as well as a more positive attitude towards English in comparison to the NK speakers. In addition, based on their dominance profiles and immersion circumstances, we assume that the KE speakers in the present study had a more extensive experience with spoken English produced by native speakers, and generally a greater amount of exposure to the language compared with the NK speakers.

### 2.2 Stimuli

Six minimal pairs that differed in voicing of the word-initial alveolar stops were used: *ten* vs. *den*, *tab* vs. *dab*, *tot* vs. *dot*, *tad* vs. *dad*, *tug* vs. *dug*, and *tub* vs. *dub*. In addition to these 12 target items, 16 English words that were composed of four sets of quadruplets containing a corner vowel (i.e., /i, æ, u, ɑ/) were included as fillers. For example, *beat, bat, boot,* and *bot*.

### 2.3 Procedures

Each participant was instructed to read each word as it appeared in isolation on the computer screen for 1.8 s, in each speaking style, i.e., casual and clear. The NE and the KE speakers performed the task in a sound-attenuated booth, and their audio was captured using a Shure KSM32 microphone which was connected to a TubeMP amplifier at a sampling rate of 44.1 kHz. The NK speakers performed the same task in a quiet classroom at a Korean University. Their recordings were made using the Marantz PMD 660 recorder and a Shure BETA 54 headset microphone, at the same sampling rate.

In order to elicit casual speech, all participants were instructed to read the words as if their interlocutors were friends or family members. For clear speech, they read each word as if they were talking to hearing-impaired or elderly interlocutors (Ferguson and Kewley-Port, 2002; Kato and Baese-Berk, 2022; Jung and Dmitrieva, 2023). Casual speech production preceded clear speech (e.g., Picheny et al., 1986; Ferguson and Kewley-Port, 2002; Smiljanic and Bradlow, 2005), and a short self-timed break was provided between the two speaking styles. Within each speaking style, words appeared in a randomized order, with three repetitions of each word.

Upon the completion of each recording session, participants were given a link to the BLP questionnaire to be completed online and a monetary reward either in cash ($5 for the NE speakers and $10 for the KE speakers) or as a Starbucks gift card (in Korean currency equivalent of $10 for the NK speakers).

### 2.4 Acoustic measurements

VOT (in milliseconds) of the word-initial stops and onset f0 of the vowels following the word-initial stops were measured for each target word in Praat (Boersma and Weenink, 2005) using custom Praat scripts. VOT was measured from the onset of aperiodic stop release and until the onset of periodic glottal pulsing, coinciding with the onset of the following vowel in cases of for positive VOT (Lisker and Abramson, 1964). A small percentage of phonologically voiced stops was produced with prevoicing or negative VOT, as is commonly observed for American English word-initial voiced stops (Dmitrieva et al., 2015). In these stops, glottal pulsing occurs during the closure, and VOT is measured backwards from the onset of release until the onset of glottal pulsing of the closure. Jung and Dmitrieva (2023) report on the analysis of negative VOT in the clear and casual speech of the NE and KE participants. However, due to a low frequency of such prevoiced stops in the Korean-accented English speech (only 7% of voiced stops produced by Korean speakers were prevoiced in the present study), we decided to exclude them from the analysis reported here.^4^ Therefore, only voiced stops produced with positive VOT (86% of voiced stops produced by NE group and 93% of voiced stops produced by both Korean groups) were included in the analysis.

Onset f0 was measured at the first instance of the vowel following the stop where Praat algorithm detected pitch. In order to eliminate the effects of individual variability, including anatomic differences, on f0 production, onset f0 values (in hertz, initially) were normalized to semitones (in ST) using the formula 12ln(x /individual mean f0)/ln2 (Dmitrieva et al., 2015). The resulting values after normalization indicate how much they deviated from individual means. For example, if the normalized value is positive (i.e., above zero), it means that it is above the individual f0 mean.

### 2.5 Statistical analysis

A linear mixed-effect model was carried out on each dependent variable (i.e., VOT and onset f0) in R (R Core Team, 2019) using the *lme4* (Bates et al., 2015) package. *p*-values were computed from the *lmerTest* (Kuznetsova et al., 2017) package. For every model, speaker group, speaking style, and stop type were entered as fixed effects, and each fixed effect was sum-coded. Initially, the random effects structure was built by entering participant and item as random intercepts and a by-participant random slope for speaking style. However, as the model resulted in a singularity fit warning for the onset f0 data, the random effects structure was modified by eliminating the term(s) that contributed to the warning (Brown, 2021). As a result of the adjustment, only the by-participant and by-item random intercepts were included for the onset f0 model.

The type-III ANOVA tests were additionally computed using the *car* package (Fox and Weisberg, 2019) to establish the significance of the main effects and their interactions. If there was a significant interaction that includes any three-level fixed effect (i.e., the speaker group factor in the current data: NE, KE, and NK speakers), the *emmeans* (Lenth et al., 2018) package was implemented to perform pairwise post-hoc tests. The present study reports only the interactions that include the stop type factor as these are important for determining how each stop type was produced by each speaker group and affected by each speaking style and thus are directly associated with the research question.

## 3 Results

### 3.1 VOT

The results of the statistical modeling for VOT showed that there were significant effects of stop type and speaking style: voiceless stops had longer VOT than voiced ones, as expected [*χ*^2^ (1) = 3799.86, *p* < 0.001], and clearly produced English stops had longer VOT than casually produced ones [*χ*^2^ (1) = 77.15, *p* < 0.001]. However, as will become clear from the discussion of the interactions below, the clear speech effect did not apply equally to both stop types and speaker groups (see also Appendix).

**Figure 2:**
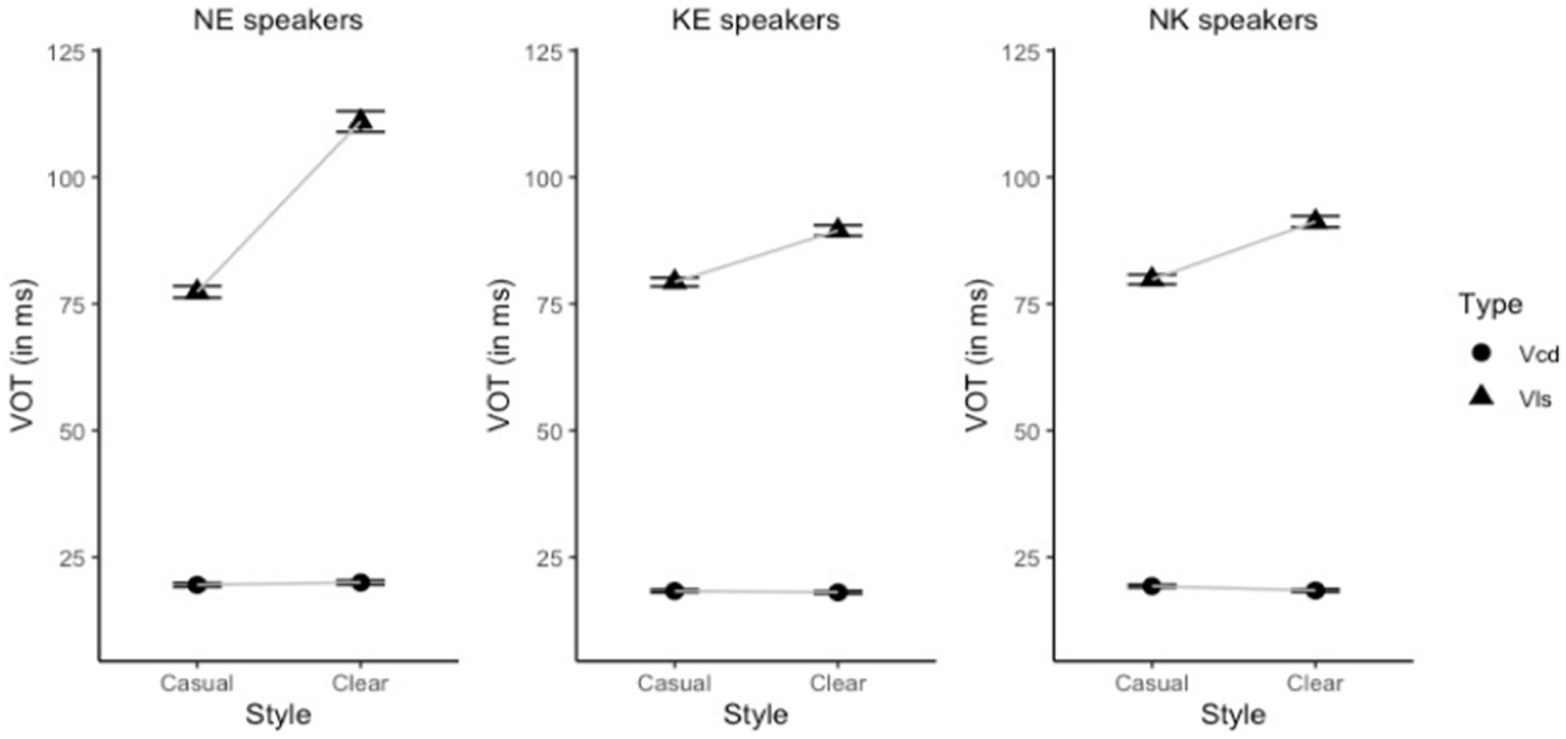
VOT of English stops (circle = voiced stops labeled as “Vcd”; triangle = voiceless stops labeled as “Vls”) produced by the NE speakers (left), the KE speakers (middle), and the NK speakers (right) in each speaking style (error bars refer to standard error).

The main effect of speaker group was not significant [*χ*^2^ (2) = 2.52, *p* = 0.283], suggesting that the speaker groups were comparable in terms of the overall VOT values. Figure 2 demonstrates the average values of VOT for English voiced and voiceless stops produced in clear and casual speaking style by the two speaker groups.

The interaction between speaker group and stop type was significant (χ2 (2) = 73.21, *p* < 0.001), indicating that there was a between-group difference in how the stop contrast was realized in terms of VOT. Table 2 provides the full results of the post-hoc analyses. As shown in Table 2, a significant speaker group by stop type interaction stemmed from the fact that NE speakers produced longer voiceless VOT in comparison with the KE and the NK speakers. In terms of VOT of voiced stops, all three groups behaved in a similar fashion. As a result, the VOT difference between voiceless and voiced stops was more pronounced for NE speakers than for the two Korean groups, although it will shortly become apparent, based on the three-way interaction, that this effect is driven by English clearly produced stops.

**Table 2:** Pairwise VOT comparisons for the effect of speaker group at each level of English stop type.

Stop type	Group contrast	Estimate	SE	*p*-value
Voiced	KE - NE	−0.90	3.51	0.964
	KE - NK	−1.16	3.13	0.927
	NE - NK	−0.26	3.51	0.99
Voiceless	KE - NE	−9.78	3.48	<0.05
	KE - NK	−1.13	3.11	0.93
	NE - NK	8.65	3.48	<0.05

The shaded cells denote the contrast that had a significant difference (*p*-value <0.05).

The interaction between speaking style and stop type was also significant [*χ*^2^ (1) = 433.75, *p* < 0.001], due to the fact that English voicing contrast was enhanced in terms of VOT in English clear speech across the speaker groups. This enhancement was achieved via an asymmetric lengthening of voiceless VOT only (see Table 3). On the other hand, VOT of voiced stops remained remarkably stable across the two speaking styles. In other words, this interaction was primarily driven by the modification of VOT of voiceless stops: a follow-up linear mixed effect model run only for voiced stops demonstrated that speaking style did not significantly affect VOT of voiced stops (*β* = 0.47, SE = 0.35, *p* = 0.19).

**Table 3:** Mean VOT duration of English voiced and voiceless stops in each speaking style.

Speaking style	Stop type	VOT (in ms)	Difference (voiceless – voiced; in ms)
Casual	Voiced	19.0	60.0
	Voiceless	79.0	
Clear	Voiced	18.6	76.9
	Voiceless	95.5	

Finally, a three-way significant interaction between speaker group, stop type, and speaking style was observed [*χ*^2^ (2) = 120.89, *p* < 0.001]. Table 4 presents the set of comprehensive pairwise VOT comparisons testing the effect of speaking style at each level of speaker group and stop type. It confirms that the effect of speaking style was significant only for voiceless stops in each speaker group. Additionally, this effect was more pronounced for the NE speakers than it was for the KE and the NK speakers, as indicated by the greater estimate value of the NE speakers. That is, every speaker group succeeded in lengthening VOT of voiceless stops in English clear speech and enhanced the VOT difference between voiced and voiceless stops as a result. However, the degree of the VOT enhancement was greater for the NE speakers (*β* = −33.62), as they lengthened VOT of voiceless stops to a greater extent in comparison with the two Korean speaker groups (KE speakers: *β* = −10.16; NK speakers: *β* = −11.41). Indeed, another pairwise comparison (see the last three rows of Table 5) showed that the difference between the three groups was confined to the VOT of voiceless stops produced in clear speaking style, as can also be observed in Figure 2. VOT of clear voiceless stops produced by the NE speakers was significantly longer than VOT of clear voiceless stops produced by both the KE and the NK speakers. However, there was no difference between the two Korean groups.

**Table 4:** Pairwise VOT comparisons for the effect of speaking style at each level of speaker group and English stop type.

Speaker group	Stop type	Style contrast	estimate	SE	*p-*value
NE	Voiced	Casual-Clear	0.07	2.32	0.976
	Voiceless	Casual-Clear	−33.62	2.17	<0.001
KE	Voiced	Casual-Clear	0.19	1.82	0.916
	Voiceless	Casual-Clear	−10.16	1.77	<0.001
NK	Voiced	Casual-Clear	0.26	1.82	0.887
	Voiceless	Casual-Clear	−11.41	1.77	<0.001

The shaded cells denote the contrast that had a significant difference (*p*-value <0.05).

**Table 5:** Pairwise VOT comparisons for the effect of speaker group at each level of speaking style and English stop type.

Speaking style	Stop type	Group contrast	estimate	SE	*p-*value
Casual	Voiced	KE-NE	−0.84	2.87	0.954
		KE-NK	−1.20	2.55	0.886
		NE-NK	−0.36	2.86	0.991
	Voiceless	KE-NE	1.95	2.82	0.768
		KE-NK	−0.50	2.52	0.978
		NE-NK	−2.45	2.82	0.659
Clear	Voiced	KE-NE	−0.96	4.57	0.976
		KE-NK	−1.13	4.05	0.958
		NE-NK	−0.17	4.57	0.999
	Voiceless	KE-NE	−21.51	4.50	<0.001
		KE-NK	−1.75	4.02	0.901
		NE-NK	19.76	4.50	<0.001

The shaded cells denote the contrast that had a significant difference (*p*-value <0.05).

### 3.2 Onset f0

Speaking style had a significant effect on English onset f0, with clearly produced stops associated with an increase in onset f0 [*χ*^2^ (1) = 65.01, *p* < 0.001], as expected, since clear speech is generally associated with higher average pitch. The effect of stop type was also significant as voiceless stops had higher onset f0 than voiced stops [*χ*^2^ (1) = 485.75, *p* < 0.001], also as expected, given the typical covariation between voicing and onset f0 in English. A non-significant effect of speaker group [*χ*^2^ (2) = 0.01, *p* = 0.996] indicated that all speaker groups had similar onset f0 values, overall.

**Figure 3:**
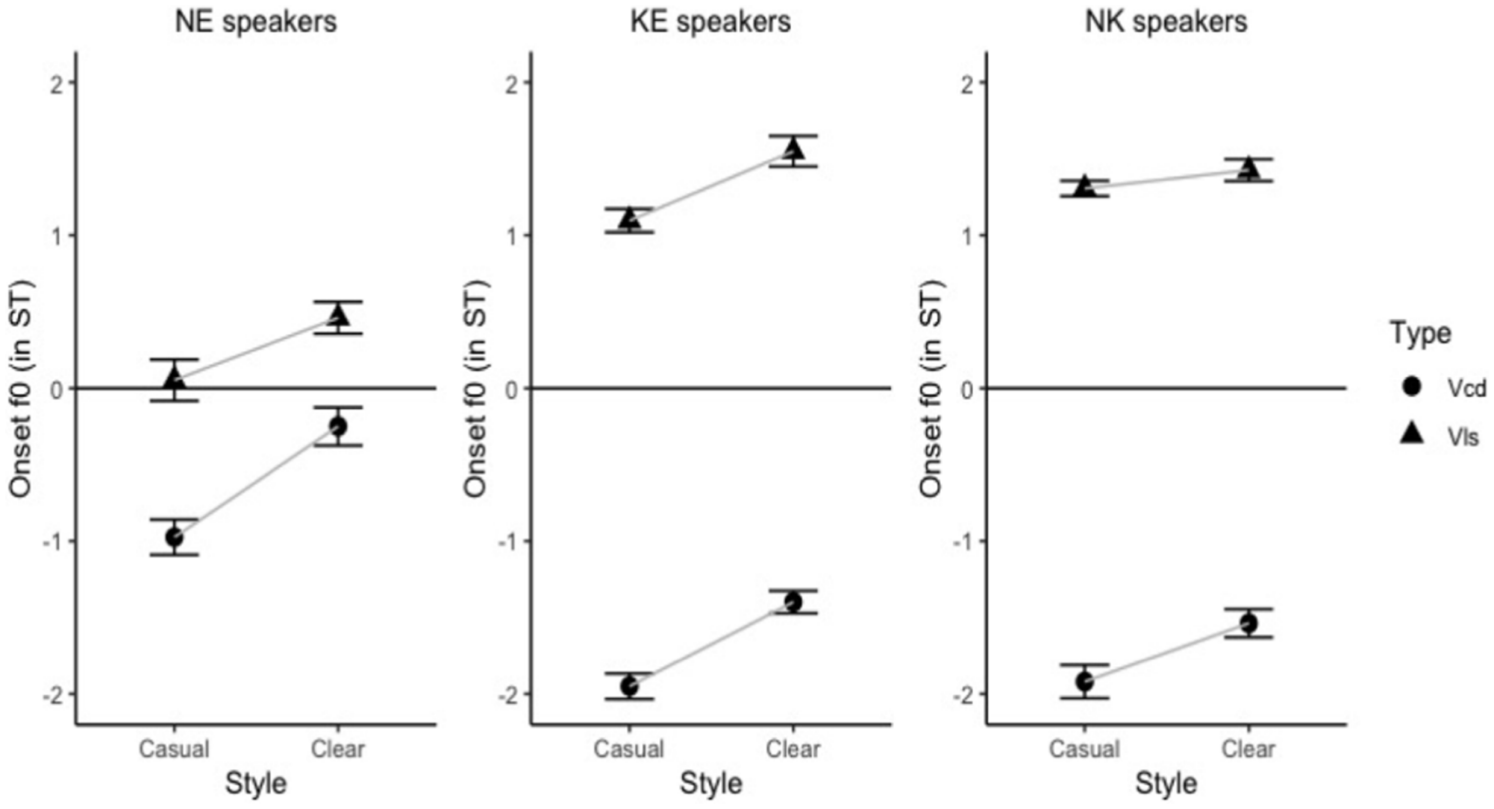
Onset f0 of English stops (circle = voiced stops labeled as “Vcd”; triangle = voiceless stops labeled as “Vls”) produced by the NE speakers (left), the KE speakers (middle), and the NK speakers (right) in each speaking style (error bars refer to standard error).

There were two significant interactions that included stop type: a speaker group by stop type interaction [*χ*^2^ (2) = 310.13, *p* < 0.001], and a speaking style by stop type interaction [*χ*^2^ (1) = 4.29, *p* < 0.05]. First, as illustrated in Figure 3, onset f0 of voiceless stops produced by the KE and the NK speakers had consistently higher values than onset f0 of voiceless stops produced by the NE speakers. An opposite pattern emerged for onset f0 of voiced stops: the NE speakers showed higher onset f0 values than the KE and the NK speakers. As a result of this discrepancy, the English voicing contrast in terms of onset f0 was greater for the KE and the NK speakers than for the NE speakers. Indeed, as shown in Table 6, these between-group differences (NE vs. KE and NK) observed for each stop type reached statistical significance, explaining the presence of a significant two-way interaction between speaker group and stop type.

**Table 6:** Pairwise onset f0 comparisons for the effect of speaker group at each level of English stop type.

Stop type	Group contrast	Estimate	SE	*p-*value
Voiced	KE-NE	−1.06	0.10	<0.001
	KE-NK	0.05	0.09	0.810
	NE-NK	1.12	0.10	<0.001
Voiceless	KE-NE	1.07	0.10	<0.001
	KE-NK	−0.04	0.09	0.875
	NE-NK	−1.11	0.10	<0.001

The shaded cells denote the contrast that had a significant difference (*p*-value <0.05).

A significant interaction between speaking style and stop type suggests that the onset f0 difference between voiced and voiceless stops was impacted by speaking style. However, contrary to expectations, the onset f0 difference was greater in casual speech (2.60 ST) than in clear speech (2.39 ST). That is, the onset f0 distance between the two stop types unexpectedly decreased in clear speech unlike VOT distance, which increased in clear speech. Therefore, there was a reversed effect of speaking style on the English onset f0 distinction between the two stop types, though the effect of speaking style itself was significant for each of the stop types. That is, onset f0 increased in clear speech for both voiced and voiceless stops (see Table 7), enhancing the overall speech signal, but not the contrast between the voicing categories. Since the three-way interaction between speaker group, speaking style, and stop type was not significant (*p* = 0.69), we conclude that speaker groups behaved in a comparable manner with respect to decreasing onset f0 difference in clear compared to casual speech.

**Table 7:** Pairwise onset f0 comparisons for the effect of speaking style at each level of English stop type.

Stop type	Style contrast	Estimate	SE	*p-*value
Voiced	Casual-Clear	−0.55	0.08	<0.001
Voiceless	Casual-Clear	−0.33	0.08	<0.001

The shaded cells denote the contrast that had a significant difference (*p*-value <0.05).

## 4 Discussion

This study investigated how L2- and L1-immersed native Korean speakers produced clear speech in their L2 (i.e., English), focusing on the enhancement of the language-specific laryngeal contrasts. We predicted that L1-based clear speech strategies could affect clear speech production in L2, especially for participants with no immersive L2 experience. We specifically predicted that such participants would exemplify a greater enhancement of onset f0 properties of voicing categories in English than L2-immersed speakers, and, naturally, than native English speakers. To verify this hypothesis, a comparison was made between native English clear speech and English clear speech produced by the two Korean speaker groups.

First, the effect of speaking style was robust, indicating that clear speech was acoustically distinct from casual speech with respect to both VOT and onset f0 as correlates of English laryngeal categories. In particular, clear speech was characterized by longer VOT and higher f0, in line with the findings from previous literature (e.g., Picheny et al., 1986; Krause and Braida, 2004; Kang and Guion, 2008). However, there was an important difference in the way these parameters were modified across voicing categories. Specifically, the overall increase in VOT duration was due exclusively to the lengthening of the VOT of voiceless stops. Furthermore, this VOT modification was consistent across participant groups, but especially pronounced in the NE group. VOT of voiced stops was remarkably stable both across groups and across speaking styles. Thus, this asymmetric manipulation of VOT in clear speech resulted in an enhanced VOT difference between voiced and voiceless stops in clear speech. In other words, voicing contrast was hyperarticulated with respect to VOT. This finding replicates earlier reports on voicing enhancement in clear speech of English monolinguals, specifically the asymmetric increase in long lag VOT of voiced stops combined with lack of changes in short lag VOT of voiced stops (e.g., Picheny et al., 1986; Smiljanic and Bradlow, 2011). Short lags’ resistance to further compression is a well-known property, and has been shown for example, in work on the effect of speech rate on VOT (Kessinger and Blumstein, 1997; Oh, 2009).

This finding indicates that clear speech does not simply lead to lengthening of all temporal features, such as VOT in the present study. Rather, clear speech strategies appear to be “guided by the principle of contrast enhancement.” (Granlund et al., 2012, p. 518). Indeed, a similar pattern was reported in Granlund et al. (2012): the VOT of the English /p/ increased while that of the English /b/ decreased in clear speech. However, we cannot completely exclude the possibility that a slower speaking rate of clear speech could have contributed to increasing VOT values to a certain degree (Miller et al., 1986).

As a result of this modification, the phonological contrast that exists in English was phonetically enhanced in clear speech, however, along the dimension of VOT only. One unexpected result was the “reverse” effect of speaking style observed for English onset f0, whereby the difference between voiced and voiceless stops actually decreased in clear speech compared to casual speech. The slightly disproportionate raising of onset f0 after voiced stops compared to raising after the voiceless ones was likely what contributed to the decreased contrast. These findings highlight the fact that VOT acts as the primary correlate of the English voicing contrast, as it carried the brunt of the enhancement work, while the preference for higher overall f0 in English clear speech appeared to have prevailed over its role as a cue to stop voicing.

In contrast to VOT, the change in onset f0 was consistent in its direction across the voicing categories, such that f0 generally increased for both voiced and voiceless stops, in accordance with the observation that clear speech is characterized by heightened pitch. It is possible that a general increase of mean f0 in clear speech is what led to the increased onset f0 of both voiced and voiceless stops. While the degree of increase was not perfectly equivalent for voiced and voiceless stops, these discrepancies (the slightly disproportionate raising of onset f0 after voiced stops compared to raising after the voiceless ones) led to an overall narrowing rather than widening of the onset f0 gap between the categories in clear speech. In other words, voicing contrast was hypoarticulated with respect to onset f0 in clear speech, and the lack of onset f0 enhancement was observed in all speaker groups. To summarize, all talker groups modified their acoustic segmental features of stops by increasing VOT for voiceless stops and increasing onset for voiced and voiceless stops, although this did not lead to greater distance in onset f0 between voiced and voiceless stops.

Another notable finding is that production and enhancement of the voicing contrast emerged as relatively independent domains in English clear speech produced by the two Korean speaker groups, as it pertains to the effect of L1 clear laryngeal phonetics. That is, both the KE and the NK speakers produced a significantly greater onset f0 difference between English voiced and voiceless stops than NE speakers did, which can be regarded as evidence of L1 influence, given that a subset of Korean laryngeal distinctions (i.e., the aspirated-lenis contrast) is signaled by pronounced f0 differences. As there were no group-based differences for this effect, it appears that the effect of L1 was consistent across groups, instead of being modulated by non-native speakers’ L2 immersive experience.

Nevertheless, neither the KE nor the NK speakers enhanced the onset f0 difference in English clear speech. In this respect, Korean speakers acted similarly to NE speakers who also did not enhance (but rather reduced) the onset f0 difference in clear speech. Interestingly, this was true for both the L2-immersed KE group and the L1-immersed NK group, whose familiarity with English was presumably less extensive. It is not clear yet why such a discontinuity between transferring production, but not enhancement strategies from L1, was found in our data. It should be noted, however, that this finding is largely in line with the results of Kato and Baese-Berk (2022, 2024), as well as Jung and Dmitrieva (2023), both reporting a tendency towards an equal or lower magnitude of acoustic enhancement in non-native than in native clear speech. Thus, the lack of onset f0 enhancement in Korean speakers’ English clear speech could have been due to this general limitation rather than to their mastery of English enhancement strategies.

An additional noteworthy finding is that both NK and KE speakers enhanced the VOT properties of English voicing contrast in clear speech in the manner comparable to NE speakers. That is, Korean speakers lengthened the voiceless VOT exclusively. However, a smaller magnitude of VOT lengthening was consistently observed in Korean-accented English clear speech compared to native English clear speech, regardless of the Korean speaker groups. There are two possible explanations for this pattern. One, given that in native Korean clear speech VOT lengthening was also relatively modest in magnitude compared to English (Kang and Guion, 2008), this finding could be interpreted as an instance of L1 influence. However, strangely, this seemingly L1-based strategy was not modulated by the degree of experience with English. If the KE speakers’ greater familiarity with English had impacted their productions of English clear speech, they would have behaved in a more native-like manner and lengthened the VOT of voiceless stops to the extent more similar to NE speakers. Contrary to this prediction, KE speakers were not different from NK speakers in the degree of voiceless VOT lengthening in clear English speech.

A second possible explanation for this pattern, which agrees with the results of Kato and Baese-Berk (2022, 2024) is that all non-native clear speech is subject to lesser magnitudes of enhancement, especially for lower proficiency speakers. While we observed a lesser magnitude of VOT enhancement for both non-native groups, there was no expected effect of L2-immersion, thus only partially aligning with the findings of Kato and Baese-Berk (2022, 2024).

The lack of differences between the L2 clear speech of the two Korean groups is puzzling, given their divergent experience with English immersion and their distinct English dominance characteristics, including proficiency and attitudes, assessed via the BLP. One possibility is that they were, in fact, not as different as we assumed. English education is early and pervasive in South Korea, and the use of English is wide-spread in media and pop-culture (e.g., Bae and Park, 2020). Thus, the purported lack of English immersion for L1-immersed speakers was possibly not entirely accurate. In fact, their dominance profiles, while distinct, were not dramatically different.

In addition, the KE group, despite continuing L2 immersion, may have reached a plateau in the development of this specific aspect of their L2 speech. In support of this possibility, results of a longitudinal study by Munro and Derwing (2008) suggest that rapid phonetic learning takes place during the initial immersion period, but plateaus after a few months. This would explain the divergence between our findings and those of Kato and Baese-Berk, who did find an effect of L2 proficiency on the magnitude of acoustic modifications in L2 clear speech. These contrastive findings highlight the importance role that specific bilingual populations play in the outcomes of research on L2 speech.

Alternatively, our specific acoustic measurements may not have captured the difference that is present in other acoustic dimensions. It is possible that the effect of L2 experience would manifest itself more clearly in the use of another acoustic correlate of voicing or a different contrast altogether. A more comprehensive analysis of the acoustics of the Korean speakers’ English clear speech is needed to be able to conclude that L2 experience played no role in the production of L2 clear speech by these populations.

A related possibility is that the enhancement of English laryngeal contrasts did not pose a great challenge to either of Korean speaker groups, despite some language-specific differences in its phonetic implementation. In other words, it is possible that Korean speakers’ familiarity with the relevant acoustic correlates of laryngeal contrasts allowed them to manipulate these correlates in clear speech to a similar extent regardless of the level of familiarity with spoken English and the language dominance profile.

## 5 Conclusion

The results of the study indicate that Korean speakers of English, both L2-immersed late sequential bilinguals and L1-immersed speakers, diverged from native English speakers in some aspects of voicing production in English clear speech. Specifically, Korean speakers lengthened voiceless VOT in clear speech, thereby increasing the acoustic distance between voiced and voiceless stops, but they did so to a smaller extent than NE speakers. In addition, Korean speakers realized a greater onset f0 difference between voiced and voiceless stops than NE speakers did, as expected based on the assumption that L1 laryngeal phonetics affects L2 production. However, this difference was not further enhanced in their clear speech. On the contrary, it was somewhat minimized, due to a slightly asymmetric pitch raising across voicing categories. Nevertheless, onset f0 difference remained relatively hyperarticulated in Korean speech compared to English speech, regardless of the speaking style. Overall, the results fit within the pattern of equal or lesser magnitude of acoustic modification in non-native clear speech compared to native clear speech.

Importantly, there were no noticeable differences between the two Korean groups that could be attributed to their distinct experiences with L2-immergion and their distinct language dominance profiles. This unexpected finding, which contrasts with some previous results (Kato and Baese-Berk, 2022, 2024) could potentially be attributed to three different causes. One, the specifics of the population: L1-immersed speakers may have had a greater degree of exposure to authentic spoken English than the lack of residence in English-dominant environment suggested. Two, while we focused on VOT and onset f0, other acoustic properties may reflect the effect of L2 experience and dominance in clear speech production more strongly. And three, production of English laryngeal categories may be a task easy enough for Korean speakers such that a certain threshold of English proficiency, achievable even in the context of L1-immersion, is sufficient to reach the mastery comparable to that achieved in an L2-immersion context. Future research should expand the range of language pairs, phonological contrasts, and acoustic parameters studied when addressing the question of bilingual clear speech in order to explore these possibilities.

## Data availability statement

The raw data supporting the conclusions of this article will be made available by the authors, without undue reservation.

## Ethics statement

The studies involving humans were approved by Purdue University Institutional Review Board. The studies were conducted in accordance with the local legislation and institutional requirements. The participants provided their written informed consent to participate in this study.

## Author contributions

YJ: Conceptualization, Formal analysis, Investigation, Methodology, Software, Visualization, Writing – original draft. OD: Conceptualization, Formal analysis, Methodology, Resources, Supervision, Writing – review & editing.

## Footnotes

^1^Rogers et al. (2010) also considered a similar factor in their study, but they focused only on the perceptual effects of clear speech produced by late Spanish learners of English and did not include acoustic analyses.

^2^Same participants as in Jung and Dmitrieva (2023).

^3^This score was obtained following the BLP scoring instructions (https://sites.la.utexas.edu/bilingual/scoring-and-interpreting-the-results/).

^4^In previous work we also observed an increase in the incidence of prevoicing in clear speech compared to casual speech: 10% of voiced stops were produced in native English casual speech and 18% in clear speech. In Korean-accented English speech, the proportion was 6% in casual speech and 8% in clear speech.
