# Supplementary material for: Enhancement of laryngeal contrasts in non-native English clear speech: a comparison between L2-immersed sequential bilinguals and L1-immersed speakers
Source: Front Hum Neurosci. 2024 Sep 13;18:1413886. doi: 10.3389/fnhum.2024.1413886 (PMC11428047; doi:10.3389/fnhum.2024.1413886)
Supplement: Supplementary file 1 [file Table_1.docx]

**Appendix: Full results of the LME models**

VOT

|  | Estimates | Std. Error | df | t value | Pr (> \|t\|)) |
| --- | --- | --- | --- | --- | --- |
| (Intercept) | 53.444 | 1.454 | 82.821 | 36.755 | < 2e-16 *** |
| Group1 | -2.162 | 1.853 | 76.621 | -1.167 | 0.247 |
| Group2 | 3.178 | 2.058 | 76.902 | 1.545 | 0.127 |
| Style1 | -9.113 | 1.038 | 75.338 | -8.783 | 3.72e-13 *** |
| Type1 | -69.158 | 1.122 | 10.573 | -61.643 | 7.31e-15 *** |
| Group1:Style1 | 4.128 | 1.411 | 74.692 | 2.926 | 0.005 ** |
| Group2:Style1 | -7.664 | 1.575 | 76.499 | -4.866 | 5.96e-06 *** |
| Group1:Type1 | 2.948 | 0.604 | 5101.467 | 4.878 | 1.10e-06 *** |
| Group2:Type1 | -5.931 | 0.693 | 5108.152 | -8.556 | < 2e-16 *** |
| Style1:Type1 | 18.569 | 0.892 | 5158.192 | 20.827 | < 2e-16 *** |
| Group1:Style1:Type1 | -8.215 | 1.201 | 5158.440 | -6.842 | 8.70e-12 *** |
| Group2:Style1:Type1 | 15.115 | 1.377 | 5158.902 | 10.977 | < 2e-16 *** |

Signif. codes: 0 ‘***’ 0.001 ‘**’ 0.01 ‘*’ 0.05 ‘.’ 0.1 ‘ ’ 1

Onset f0

|  | Estimates | Std. Error | df | t value | Pr (> \|t\|)) |
| --- | --- | --- | --- | --- | --- |
| (Intercept) | -0.178 | 0.053 | 10.2 | -3.388 | 0.007 ** |
| Group1 | 0.003 | 0.037 | 5735 | 0.067 | 0.946 |
| Group2 | 0.000 | 0.041 | 5735 | 0.012 | 0.991 |
| Style1 | -0.440 | 0.055 | 5735 | -8.063 | 9.02e-16 *** |
| Type1 | -2.319 | 0.105 | 10.2 | -22.040 | 6.18e-10 *** |
| Group1:Style1 | -0.063 | 0.074 | 5735 | -0.841 | 0.400 |
| Group2:Style1 | -0.126 | 0.082 | 5735 | -1.524 | 0.127 |
| Group1:Type1 | -0.677 | 0.074 | 5735 | -9.105 | < 2e-16 *** |
| Group2:Type1 | 1.451 | 0.082 | 5735 | 17.593 | < 2e-16 *** |
| Style1:Type1 | -0.226 | 0.109 | 5735 | -2.072 | 0.038 * |
| Group1:Style1:Type1 | 0.128 | 0.149 | 5735 | 0.863 | 0.388 |
| Group2:Style1:Type1 | -0.091 | 0.165 | 5735 | -0.554 | 0.580 |

Signif. codes: 0 ‘***’ 0.001 ‘**’ 0.01 ‘*’ 0.05 ‘.’ 0.1 ‘ ’ 1
